# The Role of Psychological Factors and Resilience in Mediating Sports Passion in Triathletes

**DOI:** 10.3390/ejihpe14120193

**Published:** 2024-11-23

**Authors:** Francesca Ancarani, Óscar Gavín-Chocano, David Molero, Germán Vicente-Rodríguez

**Affiliations:** 1 EXER-GENUD Growth, Exercise, Nutrition and Development, Research Group, University of Zaragoza, 50009 Zaragoza, Spain; fancarani@gmail.com; 2 Department of Education, Campus las Lagunillas s/n, University of Jaén, 23071 Jaén, Spain; ogavin@ujaen.es; 3 Collaborating Center of Sports Medicine, Aragon Agri-Food Institute-IA2-, Biomedical Research Center Network on the Pathophysiology of Obesity and Nutrition, Exercise and Health Spanish Research Network (EXERNET), Faculty of Health and Sport Science, FCSD, Ronda Misericordia 5, 22001 Huesca, Spain; 4 Department of Physiatry and Nursing, University of Zaragoza, 50009 Zaragoza, Spain

**Keywords:** health, passion, psychological characteristics, resilience, sport psychology

## Abstract

The present study aims to analyze the psychological characteristics associated with the performance of amateur athletes, exploring their relationships with key variables such as resilience, harmonious and obsessive passion, and perception of discomfort. The main objective is to establish the relationship between psychological characteristics and each of the variables used (resilience, personal competence, and acceptance of self and life), and to consider whether these are related to passion (harmonious and obsessive) through a structural equation analysis. The sample was composed of 110 persons: 87 adults (22 females, mean age 40.7 ± 9.1 years; 65 males, mean age in years 42.1 ± 11.9) and 23 adolescents (16 females, mean age 14.8 ± 1.3 years; 7 males, mean age 15.3 ± 2.1 years). Psychological characteristics were assessed using the Psychological Characteristics Related to Performance (CPRD) questionnaire, passion using the Passion Scale instrument, and resilience using the Resilience Scale (RS-14) questionnaire. The analyses were based on a structural equation model analysis (PLS-SEM). The results showed adequate coefficients of determination (*R* index^2^) and Stone–Geisser predictive relevance (*Q*^2^) for the personal competence resilience factors (*R*^2^ = 0.517; *Q*^2^ = 0.218) and acceptance of self and life (*R*^2^ =.415; *Q*^2^ = 0.231), as well as for the passion dimensions harmonious passion (*R*^2^ = 0.357; *Q*^2^ *=* 0.168) and obsessive passion (*R*^2^ = 0.085; *Q*^2^ *=* 0.034). In conclusion, a close relationship was demonstrated between psychological characteristics related to sports performance and the variables of resilience and passion (both harmonious and obsessive). In particular, it was confirmed that mental skills and stress management are linked to the dimensions of resilience, and these dimensions influence both types of passion. In addition, team cohesion and personal competence also play a crucial role in the development of passion, highlighting the importance of these factors in the assessment of sports performance and influencing the well-being of amateur athletes in their personal and sports development.

## 1. Introduction

Participation in sports offers multiple positive aspects; it can improve physical and cardiovascular fitness, strengthen muscles and improve endurance, facilitate weight loss, and help maintain a healthy body mass index, among other things [1]. Specifically, the association of sports practice with significant psychological and social benefits for health has been demonstrated in numerous studies, the most relevant being well-being, anxiety, and stress reduction [2].

Furthermore, negative consequences related to sports can also appear, with physical risks such as acute injuries [3] or overtraining injuries [4], as well as eating disorders and mental health problems related to psychological exhaustion and burnout, among others [5,6].

For these reasons, psychosocial characteristics play a fundamental role in sports. Aspects such as motivation and passion for the sport practiced are key elements that will significantly influence not only the performance of athletes but also their cognitive and affective responses in relation to their participation in the activity [7]. Other characteristics, such as stress tolerance, self-efficacy, emotion control, and moods, among others, also modulate the effects of participation in sport and influence performance [8,9,10]. On the other hand, competitive pressure and different external agents can turn sport into a controlling and obsessive activity that can seriously damage the well-being of individuals [11].

In recent decades, in particular, endurance sport events have grown in popularity [12], and today, not only elite athletes participate in long-distance events; in fact, there is extensive participation of amateur athletes, who are exposed to high physical [13] and psychological demands. However, the psychosocial and emotional characteristics related to performance and sport in this large population group have been little researched, so there is an essential gap in the adequate care of these athletes.

This increase in the participation of amateur athletes in tests of extreme physical and psychological demands makes us reflect on the importance of taking into account the types of preparation that they are adopting. In this regard, many amateur athletes train on their own, and in some cases they rely on information from the internet regarding nutrition or physical preparation recommendations, rather than hiring a qualified professional [14].

Among the endurance sports, triathlon is a relatively modern and emerging sport [15]. It is a very demanding and complex discipline, as it combines three different modalities: swimming, cycling, and running; therefore, it requires a high degree of physical endurance, technical–tactical skills, and mental strength. For this reason, psychosocial characteristics are particularly important, as triathletes have to face long distances, adverse conditions, and situations of extreme physical and mental fatigue [16], and they have to possess adaptability, flexibility, and specific psychological skills to effectively resolve the different transitions from one discipline to another [17].

In many cases, amateur athletes who decide to practice triathlon have very high intrinsically motivated expectations and tend to push themselves harder to overcome and exceed their limits, reaching very intense training loads that, in turn, would lead to increased risk of physical injury [18] but could probably also be associated with psychological problems [19]. This passion experienced by triathlon athletes can be defined as a continuum ranging from “harmonious passion”, an intense free inclination towards an activity that is important to the individual and in which they invest time and energy while maintaining balance with other aspects of life, to “obsessive passion”, an uncontrollable need to participate in the activity, fueled by feelings of social acceptance or self-esteem and leading to risks of imbalance with other aspects of life and maladaptive outcomes [20]. Both types of passion influence athletes’ performance and well-being, as well as their mood in terms of levels of positive and negative affect [19]. People with harmonious passion find greater satisfaction in their sports participation, are more resilient, and experience a greater sense of autonomy. Therefore, they feel excited and motivated (positive affect) by their dedication to triathlon. This type of participation is desirable and fits into an active and healthy lifestyle approach.

Conversely, obsessive passion can lead to stress, burnout, dependence, and lack of balance, causing them to experience high levels of negative affect, with feelings of distress and unpleasant engagement [20,21]. In the latter situation, sports performance is likely to be affected, but the direct consequences on mental health could be of even greater significance. It is not known with certainty what variables can modulate one or another response to competition in these athletes, so knowing variables associated with the person, e.g., their previous sporting experience and their performance expectations, could help us to understand how to guide practice and training to promote positive affect and a healthy and safe practice.

Sports performance can also vary according to the different stages of life in which the athlete is, due to the physical and psychological changes that they are experiencing. Adolescence, for example, can be a risk stage for sport dropout; young people go through a phase of rapid physical development and significant psychological and social changes [22] that can affect their motivation and self-esteem [23]. During this stage, they usually face a greater academic load that can lead to difficulties in reconciling sporting activity, and if there is also a lack of support or guidance from coaches, parents, or other figures in the athlete’s environment, the risk of abandonment can increase considerably [24].

As age progresses, athletes’ performance fluctuates, reaching peaks of performance followed by a gradual decline due to physiological factors [25] and other external factors, such as work–life balance [26]. Sporting experience gained over the years can compensate to some extent for the loss of physical performance in adulthood [27]. Adapting training and nutrition, as well as improving certain psychological skills, can help to maximize performance in each phase of life, as well as to preserve the well-being of individuals.

**Figure 1:**
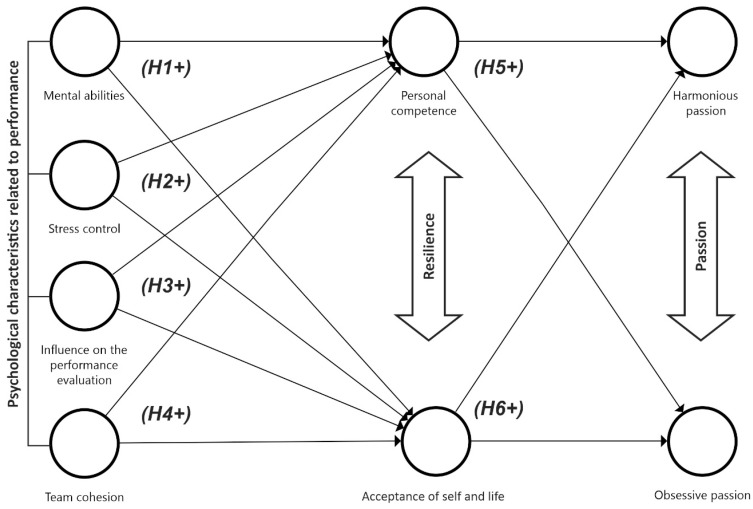
Proposed theoretical model.

Considering that more evidence is needed to establish an association between the psychological profiles of amateur athletes and their levels of well-being and performance, in general, we consider the following hypotheses (see Figure 1):

**Hypothesis** **1** **(H1).** *Mental abilities are related to each of the dimensions of resilience (personal competence and acceptance of self and life), and these act as determinants of passion (harmonious and obsessive).*

**Hypothesis** **2** **(H2).** *Stress management is related to resilience factors (personal competence and acceptance of self and life), and these enhance passion (harmonious and obsessive).*

**Hypothesis** **3** **(H3).** *Influence on the performance evaluation is related to the dimensions of resilience (personal competence and acceptance of self and life), and these act as determinants of passion (harmonious and obsessive).*

**Hypothesis** **4** **(H4).** *Team cohesion is related to resilience factors (personal competence and acceptance of self and life), and these enhance passion (harmonious and obsessive).*

**Hypothesis** **5** **(H5).** *The personal competence dimension of resilience is related to harmonious and obsessive passion.*

**Hypothesis** **6** **(H6).** *The resilience factor acceptance of self and life is related to obsessive passion.*

## 2. Materials and Methods

### 2.1. Participants

The sample consisted of 110 amateur triathletes from Zaragoza (Spain). A non-probabilistic, casual or accessibility sampling was used for their selection. The distribution of the participants by gender was as follows: 38 women (50.3%) and 72 men (65.45%). Among the participants, we found two categories according to age: 87 adults (22 women, 40.7 ± 9.1 years; 65 men, 42.1 ± 11.9 years) and 23 participants of adolescent age (16 women, 14.8 ± 1.3 years; 7 men, 15.3 ± 2.1 years). The age range of the participants was between 14 and 50 years of age. The athletes participating in the study were drawn from a diverse range of sports clubs. They competed both individually and as part of teams in the club rankings. As for the statistical power analysis, an estimate was made to achieve a power of 80%, assuming a confidence interval of 95% and a moderate–low effect size (Cohen’s *d* of 0.5), with an estimate of 120 subjects (close to the sample size of our study).

### 2.2. Instruments

The Questionnaire of Psychological Characteristics Related to Performance (CPRD) [28] is a tool used in research and in the sports field to evaluate different psychological aspects that can influence sports performance. It is composed of 5 factors and 55 items, with responses on a 5-point Likert scale with a range between “1: totally disagree” and “5: totally agree”. In our study, we employed 4 of the 5 subscales; the reliability obtained for each of them is as follows: stress control (α = 0.88), influence on performance evaluation (α = 0.78), mental skills (α = 0.71), and team cohesion (α = 0.78). The motivation subscale was not used in our study, as it is composed of fewer items.

The Passion Scale [29] comprises 14 items, of which 7 items assess obsessive passion and 7 questions assess harmonious passion, which are answered using a 5-point Likert scale ranging between “1: strongly disagree” and “5: strongly agree”. The reliability is α = 0.86 and α = 0.87 for obsessive passion and harmonious passion, respectively.

Resilience was assessed using the Resilience Scale (RS-14) [30], which measures the degree of individual resilience. The scale comprises 2 factors: personal competence (11 items) and acceptance of self and life (3 items). A 7-point Likert scale with a range between “1: strongly disagree” and “7: strongly agree” must be answered. The Cronbach’s *α* internal consistency coefficient for each factor is 0.89 for personal competence and 0.80 for acceptance of self and life.

### 2.3. Procedure

National and international ethical guidelines were followed to carry out the research and data collection, complying with Spanish legislation and legal regulations for human clinical research (Law 14/2007 on biomedical research). The participants were assured that their responses would be kept anonymous and confidential, and that all information provided would be used exclusively for scientific purposes. As there were minors among the participants, authorization was previously requested from their families, and the information obtained was kept anonymous and confidential, ensuring the ethical principles of research established in the Declaration of Helsinki by the World Medical Association [31]. The instruments were administered individually through the Google platform (Google LLC, Mountain View, California, United States) between June and July 2023. The approximate response time for each subject was 15 min. The Ethics Committee of the University of Jaén (Spain) has approved this study for research on human subjects, ID code OCT.22/2-LINE.

### 2.4. Data Analysis

Several statistical analyses were carried out in this study. A previous analysis was performed to evaluate the validity, reliability, and internal consistency of each instrument. This was achieved using a Confirmatory Factor Analysis (CFA) to verify the psychometric properties of the instruments. A multivariate hypothesis test was performed to verify the data’s normality, revealing that the distribution was not normal. All of these analyses were performed using Jamovi software version 1.2 and SmartPLS 4.

As for the coefficients considered in the study, the χ2/df ratio, the root-mean-square error of approximation (RMSEA), and the comparative fit index (CFI) were used. The model was considered adequate when the TLI and CFI were ≥0.95 and the RMSEA was close to 0.07. To assess convergent validity, the average variance extracted (AVE) was calculated, which had to be greater than 0.50, following the recommendations of Hair et al. [32]. For discriminant validity, the criteria of Fornell and Larcker [33] were applied, which indicate that the square root of the AVE of each variable should be greater than the correlations it has with the other variables. In addition, the Heterotrait–Monotrait Ratio (HTMT) was used, which had to be less than 0.90, according to Henseler [34]. To evaluate the significance, size, and direction of the coefficients of the structural model, the bootstrapping technique was used, with 5000 samples [33,35]. The results were considered to be statistically significant at a 95% confidence level (*p* < 0.05). PLS-SEM was used because of its suitability for explaining and predicting endogenous constructs [33].

## 3. Results

### PLS Path Model

**Figure 2:**
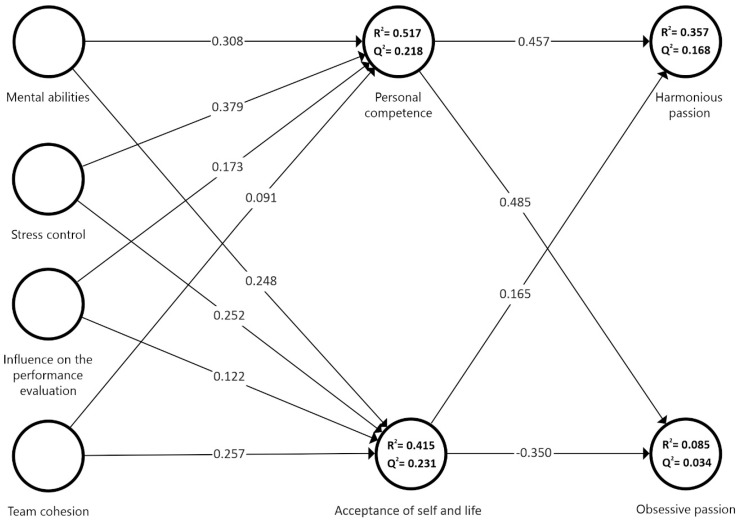
PLS path model and estimation results.

To evaluate the multicollinearity of each dimension, the variance inflation factor (VIF) test was performed, following the criteria of Becker et al. [36]. The results showed that there were no problems of collinearity. To analyze the structural model, the bootstrapping technique was used, with 5000 subsamples [37]. Standard errors and *t-statistics* of the path coefficient with 95% confidence intervals of the standardized regression coefficients were checked (see Figure 2). The coefficient of determination (*R*^2^) and cross-form predictive relevance (*Q*^2^) were analyzed, as well as the paths between variables [32].

The *R-index*^2^ values were as follows: personal competence 51.7%, acceptance of self and life 41.5%, harmonious passion 35.7%, and obsessive passion 8.5%. Therefore, these were considered to be adequate coefficients of determination according to Chin’s criteria [38]. Predictive relevance was also assessed using the Stone–Geisser *Q* statistic^2^ [32]. The results reported the following *Q* values^2^: personal competence (0.218), acceptance of self and life (0.231), harmonious passion (0.168), and obsessive passion (0.034), indicating minor predictive relevance [32].

Table 1 shows the results of the Cronbach’s alpha coefficients, external loadings, and composite reliability index (CFI) values. As for convergent validity, it was assessed by estimating the average variance extracted (AVE), where values above 0.5 are considered to be indicative of an adequate representation of the observable variable loadings; that is, a high AVE value will reflect a higher representativeness of the observed variable loadings [39].

Discriminant validity (see Table 2) was evaluated according to the criteria established by Fornell and Larcker [33] and the Heterotrait–Monotrait Ratio (HTMT). For discriminant validity to be satisfactory, the items represented in bold type on the main diagonal of Table 2 must be significantly larger than the off-diagonal items in the corresponding rows and columns, as proposed. The Heterotrait–Monotrait Ratio (HTMT) shows the difference in the latent variable of each factor relative to the others, criteria that were met in our study. All HTMT values were less than 0.85, as recommended in the literature [37].

Table 3 shows the hypothesis testing, following the criteria of Hair et al. [32], where the relationship between variables can be observed. The results of the *t*-test were obtained, where values higher than 1.96 indicate consistency of the model.

The great majority of the relationships between variables showed a value higher than the critical value of *t* and significance: acceptance of self and life -> obsessive passion (*β* = −0.350, *t* = 1.859, *p* < 0.001, CI 95% [−0.758; 0.080]); influence on the performance evaluation -> personal competence (*β* = 0.173, *t =* 1.956, *p* < 0.001, CI 95% [0.026; 0.347]); mental abilities -> acceptance of self and life (*β* = 0.248, *t* = 2.324, *p* < 0.001, CI 95% [0.036; 0.423]); mental abilities -> personal competence (*β* = 0.308, *t* = 4.122, *p* < 0.001, CI 95% [0.139; 0.437]); personal competence -> harmonious passion (*β =* 0.457, *t =* 3.322, *p* < 0.001, CI 95% [0.224; 0.755]); personal competence -> obsessive passion (*β* = 0.485, *t* = 2.964, *p* < 0.001, CI 95% [0.184; 0.892]); stress control -> acceptance of self and life (*β* = 0.252, *t =* 2.374, *p* < 0.001, CI 95% [0.052; 0.445]); stress control -> personal competence (*β* = 0.379, *t* = 4.390, *p* < 0.001, CI 95% [0.215; 0.551]); team cohesion -> acceptance of self and life (*β* = 0.257, *t* = 2.541, *p* < 0.001, CI 95% [0.086; 0.468]).

## 4. Discussion

This study analyzed how psychological characteristics related to the performance of amateur athletes are related to resilience, passion (harmonious and obsessive), and perception of discomfort. Structural equation analysis was used to understand how these variables interact.

The analysis of the results revealed significant relationships between the psychological characteristics of performance and the variables of resilience and both harmonious and obsessive passion. First, it was observed that, according to the first hypothesis (H1), mental abilities are closely related to each of the dimensions of resilience—both personal competence and acceptance of self and life. Furthermore, evidence was found that these dimensions of resilience act as determinants of the two types of passion (harmonious and obsessive). This suggests that, for the athletes analyzed in our study, resilience plays an essential role in the management of harmonious and obsessive passion, facilitating adaptation and helping to overcome the challenges associated with both forms of passion.

There are several valuable insights to be gained from an analysis of the international literature, which could be used to inform the interpretation of the results of the present study, especially those that analyze the association of resilience with sports passion—for example, the survey by Vankakova et al. [40], which stands out for its focus on the relationships between character strengths, passion, and resilience in athletes. In their research, they found that all character strength factors were positively related to resilience. Specifically, they observed that harmonious passion, not obsessive passion, acted as a partial mediator in the relationships between all character strength factors and resilience. This evidence suggests that how athletes experience their passion may influence how character strengths, such as positivity and persistence, relate to their ability to bounce back from challenges.

In our study, we found that mental skills—which include the ability to self-assess and self-regulate overall arousal levels, set goals, evaluate their own performance, and master psychological techniques such as visualization and attentional skills, among others [28]—could strengthen the dimensions of resilience (as shown by the association observed in the PLS path model), which, in turn, influences passion (harmonious and obsessive). This can be explained by the fact that mental skills provide athletes with the necessary tools to effectively face the challenges and adversities that may arise in their sports career, helping them to develop greater self-confidence. This suggests that mental skills may be an essential factor in cultivating resilience and passion in athletes.

These two studies [28,40] offer valuable insights into the relationships between mental abilities, resilience, passion, and character strengths in sports. They highlight the importance of considering individual psychological aspects (mental skills and character strengths) and emotional dimensions (resilience and passion) in sports development and performance. Furthermore, they suggest that the relationships between these factors may be complex and multidimensional, highlighting the need for future research to better understand how they interact and influence each other in the sport context.

Another critical question is how stress control is related to resilience factors (both personal competence and acceptance of self and life). We hypothesized that these are related and enhance both harmonious and obsessive passion (H2). The results obtained confirm the association between stress management and resilience factors, and that, by enhancing these factors, both harmonious and obsessive passion are strengthened.

Previous research has highlighted the critical role that resilience plays in coping with and recovering from stress in competitive sports contexts [41,42]. Resilient athletes have been found to be able to manage the pressures and demands associated with competition more effectively.

Stress management is considered to be a crucial skill for fostering resilience in athletes, as it enables them to regulate their emotions, stay focused, and make effective decisions even under pressure. In turn, resilience acts as a buffer against stress, helping athletes to face challenges with a positive and constructive attitude. These results follow the same trend as those obtained in another paper, which found that coping strategies predict 34% of variability in resilience, with active coping being the strategy with the most significant impact on resilience [43]. It appears that those who adopt this approach actively seek to address challenges by taking concrete steps to mitigate or eliminate stressors. This proactive response may give them more remarkable ability to recover from adversity, cope with trauma, and envision a promising future [43].

We have not found studies that explicitly reflect the association between stress management and resilience factors and their enhancing effects on harmonious and obsessive passion in amateur athletes. However, we should note that most research reflects a mediating effect of both types of passion on individuals’ resilience. For example, in the study of Paquette et al. [44], it was observed that, in an academic context, when individuals experience stressful situations, harmonious passion is associated with greater resilience in various areas of life. In contrast, obsessive passion is associated with less or even no resilience. Although our study does not directly address this relationship, the findings suggest a possible connection between stress management, resilience, and passion in athletes, which may be a promising area for future research.

The relationships postulated in Hypotheses 3 and 4 were also validated. For Hypothesis 3 (H3), it was found that the influence on performance evaluation is related to both dimensions of resilience, which, in turn, act as determinants of both harmonious and obsessive passion.

One possible explanation could be that the influence on performance evaluation may be influenced by how individuals perceive and process the results of their sports performance [42]. Those athletes who are self-accepting and confident in their ability to cope with obstacles may be more likely to evaluate their performance positively and constructively. In turn, this resilient attitude can fuel their passion for sport, both harmoniously and obsessively [45].

The influence on performance evaluation is a variable that has been little studied in the scientific literature. However, it has been seen that there is a negative relationship between psychological distress and stress control, as well as the influence of evaluation on performance [45], suggesting that, as psychological distress increases, the ability of athletes to effectively manage stress and positively evaluate their performance tends to decrease.

Regarding Hypothesis 4 (H4), it was confirmed that team cohesion is linked to resilience factors, and that these dimensions also enhance passion. This suggests that the emotional and motivational bonds that athletes develop with their team may significantly influence their ability to face challenges and adversities, thus strengthening their resilience and determination in pursuing their sporting goals. In terms of validating this hypothesis, the scientific literature offers us a study in which it was found that group cohesion in sports teams impacts mental toughness through the mediating effect of harmonious passion and obsessive passion [46].

This seems to indicate that the intensity and commitment of passion, both harmonious and obsessive, may influence the relationship between team cohesion and athletes’ mental toughness. These findings highlight the importance of emotional and motivational bonding within the team, highlighting its relevance when designing specific programs to improve psychological competencies.

Acceptance of Hypothesis 5 (H5) showed that the personal competence dimension of resilience, which is reflected in adequate levels of self-confidence, independence, decisiveness, resourcefulness, and perseverance, is related to harmonious and obsessive passion [41]. On the one hand, greater personal competence could boost harmonious passion by promoting a deep and healthy commitment to the sporting activity, supported by a sense of competence and self-efficacy [43]. On the other hand, the same confidence in one’s own abilities could also fuel obsessive passion by generating intense dedication and extreme commitment to the sport, sometimes even excessively so.

Hypothesis 6 (H6), states that the factor of acceptance of self and life, which includes good adaptability, flexibility, and a stable life outlook, is related to obsessive passion. The characteristics of this factor may act as catalysts for obsessive passion by allowing athletes to become deeply immersed in their sports activity, with a high degree of commitment and dedication. For example, adaptation to circumstances may drive or facilitate the development of an obsessive passion towards sports activity.

Studies exploring the antecedents of passion for an activity are scarce. Chamorro et al. [47] examined the motivational antecedents of sports passion, finding that players’ satisfaction with the three basic psychological needs predicted their harmonious and obsessive passion by mediating autonomous and controlled motivations. In particular, autonomy and competence satisfaction directly and indirectly affect both types of passion.

Although both harmonious and obsessive passion may share specific determinants, it appears that obsessive passion requires additional regulation or is more influenced by negative factors. Harmonious passion is usually associated with positively integrating the activity into the individual’s life, promoting well-being and balance. In contrast, obsessive passion may arise from a more compulsive and controlling relationship with the activity, suggesting that, in addition to positive factors such as resilience, it may also be influenced by a need for negative regulation or tighter control parameters.

This negative regulation may include stress and pressure management strategies, which act as mechanisms to prevent obsessive passion from becoming detrimental. Therefore, it is crucial to consider factors that enhance resilience and passion in general as well as those that help manage and regulate the more intense and potentially harmful aspects of obsessive passion.

These findings suggest a complex interconnection between the psychological variables studied, highlighting the importance of resilience in the relationship between mental abilities, stress control, performance evaluation, and passion in the analyzed athletes.

### Limitations

Before concluding, it is necessary to consider some limitations of this study, including the number of participants and the use of non-probabilistic sampling, which prevent us from generalizing the results to other contexts and other types of athletes. The inclusion of adolescents represents a potential limitation for the results obtained, but it provides us with the first data to explore this specific population. The fact that a gender-based analysis was not conducted is a limitation of this study that must be considered in upcoming proposals. Future research should include psychological counselling and support processes for athletes and coaches to base future interventions on the evidence obtained.

## 5. Conclusions

In conclusion, a close relationship was demonstrated between psychological characteristics related to sports performance and the variables of resilience and passion (both harmonious and obsessive). In particular, it was confirmed that mental skills and stress management are linked to dimensions of resilience, and that these dimensions influence both types of passion. In addition, team cohesion and personal competence also play a crucial role in developing passion, highlighting the importance of these factors in assessing sports performance and influencing the well-being of both professional and amateur athletes in their personal and sports development.

## Figures and Tables

**Table 1:** Correlation weights, reliability estimates, and convergent validity statistics.

	α	Composite Reliability (rho A)	Composite Reliability	Variance Extracted Mean (AVE)
Stress control	0.884	0.900	0.894	0.539
Influence on the performance evaluation	0.781	0.788	0.821	0.570
Mental abilities	0.713	0.725	0.811	0.564
Team cohesion	0.787	0.833	0.850	0.542
Obsessive passion	0.869	0.911	0.897	0.557
Harmonious passion	0.870	0.878	0.901	0.570
Personal competence	0.891	0.905	0.911	0.587
Acceptance of self and life	0.801	0.837	0.885	0.720

**Table 2:** Measurement model: discriminant validity.

Fornell–Larcker Criterion	1	2	3	4	5	6	7	8
1. Acceptance of self and life	**0.849**							
2. Harmonious passion	0.532	**0.755**						
3. Influence on the performance evaluation	0.383	0.257	**0.609**					
4. Mental abilities	0.468	0.511	0.196	**0.681**				
5. Obsessive passion	0.041	0.444	−0.156	0.308	**0.746**			
6. Personal competence	0.805	0.589	0.493	0.511	0.204	**0.698**		
7. Stress control	0.511	0.350	0.638	0.341	0.032	0.628	**0.583**	
8. Team cohesion	0.483	0.498	0.203	0.430	0.132	0.401	0.377	**0.736**
**Heterotrait–Monotrait Ratio (HTMT)**	**1**	**2**	**3**	**4**	**5**	**6**	**7**	**8**
1. Acceptance of self and life								
2. Harmonious passion	0.624							
3. Influence on the performance evaluation	0.374	0.311						
4. Mental abilities	0.583	0.619	0.284					
5. Obsessive passion	0.187	0.521	0.420	0.367				
6. Personal competence	0.936	0.655	0.493	0.611	0.248			
7. Stress control	0.495	0.407	0.820	0.414	0.291	0.605		
8. Team cohesion	0.558	0.556	0.307	0.540	0.214	0.427	0.425	

**Table 3:** Path coefficients (standardized regression coefficients).

	Path Coefficient (*β*)	Standard Deviation	*t*-Statistic	95% Bootstrap Confidence Intervals (Paths)	*p*
Acceptance of self and life -> Harmonious passion	0.165	0.176	0.936	[−0.241; 0.423]	0.350
Acceptance of self and life -> Obsessive passion	−0.350	0.211	1.859	[−0.758; 0.080]	***
Influence on the performance evaluation -> Acceptance of self and life	0.122	0.104	1.165	[−0.042; 0.344]	0.245
Influence on the performance evaluation -> Personal competence	0.173	0.088	1.956	[0.026; 0.347]	***
Mental abilities -> Acceptance of self and life	0.248	0.107	2.324	[0.036; 0.423]	***
Mental abilities -> Personal competence	0.308	0.075	4.122	[0.139; 0.437]	***
Personal competence -> Harmonious passion	0.457	0.137	3.332	[0.224; 0.755]	***
Personal competence -> Obsessive passion	0.485	0.164	2.964	[0.184; 0.892]	***
Stress control -> Acceptance of self and life	0.252	0.106	2.374	[0.052; 0.445]	***
Stress control -> Personal competence	0.379	0.086	4.390	[0.215; 0.551]	***
Team cohesion -> Acceptance of self and life	0.257	0.101	2.541	[0.086; 0.468]	***
Team cohesion -> Personal competence	0.091	0.084	1.085	[−0.049; 0.279]	0.278

*** = *p* < 0.001.

## Data Availability

The datasets generated during and/or analyzed during the current study are available from the corresponding author upon reasonable request.

## References

[1] Bull F.C., Al-Ansari S.S., Biddle S., Borodulin K., Buman M.P., Cardon G., Carty C., Chaput J.P., Chasin S., Chou R. (2020). World Health Organization 2020 guidelines on physical activity and sedentary behaviour. Br. J. Sports Med..

[2] Singh B., Olds T., Curtis R., Dumuid D., Virgara R., Watson A., Szeto K., O’Connor E., Ferguson T., Eglitis E. (2023). Effectiveness of physical activity interventions for improving depression, anxiety and distress: An overview of systematic reviews. Br. J. Sports Med..

[3] Ristolainen L., Toivo K., Parkkari J., Kokko S., Alanko L., Heinonen O.J., Korpelainen R., Savonen K., Selänne H., Vasankari T. (2019). Acute and overuse injuries among sports club members and nonmembers: The Finnish Health Promo/ng Sports Club (FHPSC) study. BMC Musculoskelet. Disord..

[4] Walters B.K., Read C.R., Estes A.R. (2018). The effects of resistance training, overtraining, and early specialization on youth athlete injury and development. J. Sports Med. Phys. Fit..

[5] Groenewal P.H., Putrino D., Norman M.R. (2021). Burnout and Motivation in Sport. Psychiatr. Clin. N. Am..

[6] Jordalen G., Lemyre P.N., Solstad B.E., Ivarsson A. (2018). The Role of Self-Control and Motivation on Exhaustion in Youth Athletes: A Longitudinal Perspective. Front. Psychol..

[7] St-Cyr J., Vallerand R.J., Chénard-Poirier L.A. (2021). The Role of Passion and Achievement Goals in Optimal Functioning in Sports. Int. J. Environ. Res. Public Health.

[8] Brace A.W., George K., Lovell G.P. (2020). Mental toughness and self-efficacy of elite ultra-marathon runners. PLoS ONE.

[9] Gameiro N., Rodrigues F., Antunes R., Matos R., Amaro N., Jacinto M., Monteiro D. (2023). Mental Toughness and Resilience in Trail Runner’s Performance. Percept. Mot. Ski..

[10] Méndez-Alonso D., Prieto-Saborit J.A., Bahamonde J.R., Jiménez-Arberás E. (2021). Influence of Psychological Factors on the Success of the Ultra-Trail Runner. Int. J. Environ. Res. Public Health.

[11] Souter G., Lewis R., Serrant L. (2018). Men, Mental Health and Elite Sport: A Narrative Review. Sports Med. Open.

[12] Nikolaidis P., Knechtle B. (2018). Age of peak performance in 50-km ultramarathoners-is it older than in marathoners?. Open Access J. Sports Med..

[13] Calleja-Romero A., López-Laval I., Sitko S., Hernando D., Vicente-Rodríguez G., Bailón R., Garatachea N. (2020). Effects of a 75-km mountain ultra-marathon on heart rate variability in amateur runners. J. Sports Med. Phys. Fit..

[14] Kopetschny H., Rowlands D., Popovich D., Thomson J. (2018). Long-Distance Triathletes’ Intentions to Manipulate Energy and Macronutrient Intake Over a Training Macrocycle. Int. J. Sport Nutr. Exerc. Metab..

[15] Triathlon Participation in the, U.S. 2010–2021. https://www.statista.com/statistics/191339/participants-in-triathlons-in-the-us-since-2006/.

[16] Vleck V., Millet G.P., Alves F.B. (2014). The impact of triathlon training and racing on Athletes’ General Health. Sports Med..

[17] Olmedilla A., Torres-Luque G., García-Mas A., Rubio V.J., Ducoing E., Ortega E. (2018). Psychological Profiling of Triathlon and Road Cycling Athletes. Front. Psychol..

[18] León-Guereño P., Tapia-Serrano M.A., Sánchez-Miguel P.A. (2020). The relationship of recreational runners’ motivation and resilience levels to the incidence of injury: A mediation model. PLoS ONE.

[19] Mylonopoulos N., Theoharakis V. (2023). Passion for an activity and its role on affect: Does personality and the type of activity matter?. Front. Psychol..

[20] Méndez-Giménez A., Cecchini-Estrada J.A., Fernández-Río J. (2017). Passion, Self-Determined Motivation and Self-Regulation of Learning in Sport. Iberoam. J. Psychol. Diagn. Eval..

[21] Boucher V.G., Caru M., Martin S.M., Lopes M., Comtois A.S., Lalonde F. (2021). Psychological Status During and After the Preparation of a Long-distance Triathlon Event in Amateur Athletes. Int. J. Exerc. Sci..

[22] Best O., Ban S. (2021). Adolescence: Physical changes and neurological development. Br. J. Nurs..

[23] Golovey L.A., Danilova M.V., Gruzdeva I.A., Rykman L.V. (2021). Psychological Well-Being and Intra-personal Conflicts in Adolescence. Psychol. Russ. State Art.

[24] Aunola K., Sorkkila M., Viljaranta J., Tolvanen A., Ryba T.V. (2018). The role of parental affection and psychological control in adolescent athletes’ symptoms of school and sport burnout during the transition to upper secondary school. J. Adolesc..

[25] Willy R.W., Paqueqe M.R. (2019). The Physiology and Biomechanics of the Master Runner. Sports Med. Arthrosc. Rev..

[26] González-Fernández M.D., Selva-Olid C., Torregrosa M. (2018). Women and female referees: Life stories of a double minority in sport. J. Sport Psychol..

[27] Lepers R., Rüst C.A., Stapley P.J., Knechtle B. (2013). Relative improvements in endurance performance with age: Evidence from 25 years of Hawaii Ironman racing. Age.

[28] Gimeno F., Buceta J.M., Pérez-Llanta M.D.C. (2012). The Psychological Characteristics Related to Sport Performance (CPRD) questionnaire: Psychometric characteristics. Análise Psicológica.

[29] Pedrosa I., García-Cueto E., Torrado J., Arce C. (2017). Spanish adaptation of the Passion Scale to the sport domain. Rev. Iberoam. Diagnóstico Eval. Psicol..

[30] Sánchez-Teruel D., Robles-Bello M.A. (2015). 14-item Resilience Scale (RS-14): Psychometric Properties of the Spanish Version. Rev. Iberoam. Diagnóstico Evaluación Avaliação Psicológica.

[31] WMA Declaration of Helsinki. Ethical Principles for Medical Research on Human Beings. Proceedings of the 64th General Assembly.

[32] Hair J.F., Sarstedt M., Ringle C.M., Gudergan S.P., Castillo-Apraiz J., Cepeda-Carrión G.A., Roldán J.L. (2021). Advanced Manual of Partial Least Squares Structural Equation Modeling (PLS-SEM).

[33] Fornell C., Larcker D.F. (1981). Evaluating structural equation models with unobservable variables and measurement error. J. Mark. Res..

[34] Henseler J. (2017). Bridging design and behavioral research with variance-based structural equation modeling. J. Advert..

[35] Kline R. (2015). Principles and Practice of Structural Equation Modeling.

[36] Becker J.M., Ringle C.M., Sarstedt M., Völckner F. (2015). How collinearity affects mixture regression results. Mark. Lett..

[37] Henseler J., Ringle C.M., Sinkovics R.R., Sinkovics R.R., Ghauri P.N. (2009). The use of partial least squares path modeling in international marketing. Advances in International Marketing.

[38] Chin W.W., Marcoulides G.A. (2013). The partial least squares approach to structural equation modeling. Modern Methods for Business Research.

[39] Becker J.M., Ringleb C.M., Sarstedtc M. (2018). Estimating moderating effects in PLS-SEM and PLSc-SEM: Interaction term generation data treatment. J. Appl. Struct. Equ. Model..

[40] Vankakova J., Chamarro A., Martínez- Martí M.L. (2021). Does Passion mediate the Effect of Character Strengths on the Resilience of Athletes?. Cuad. Psicol. Deporte.

[41] Codonhato R., Rubio V., Oliveira P.M.P., Resende C.F., Rosa B.A.M., Pujals C., Fiorese L. (2018). Resilience, stress and injuries in the context of the Brazilian elite rhythmic gymnastics. PLoS ONE.

[42] García-Secades X., Molinero O., Ruíz-Barquín R., Salguero A., De La Vega R., Márquez S. (2017). Resilience and recovery-stress in competitive athletes. Cuad. Psicol. Deporte.

[43] Morán-Astorga M.C., Finez-Silva M.J., Menezes dos Anjos E., Pérez-Lancho M.C., Urchaga-Litago J.D., Vallejo-Pérez G. (2019). Coping strategies that predict greater resilience. INFAD J. Psychol. Int. J. Dev. Educ. Psychol..

[44] Paquette V., Holding A.C., Cimon-Paquet C., Giroux A., Boucher V.B., Vallerand R.J. (2023). Pursuing, developing, and letting go of a passionate activity when facing adversity during a pandemic: Associations with well-being and ill-being. Personal. Individ. Differ..

[45] Lorenzón J.I., González Insua F., Aceiro M.A., Delfino G. (2022). Psychological distress related to psychological skills associated with sports performance in young athletes. Cienc. Psicológicas.

[46] Gu S., Bi S., Guan Z., Fang X., Jiang X. (2022). Relationships among Sports Group Cohesion, Passion, and Mental Toughness in Chinese Team Sports Athletes. Int. J. Environ. Res. Public Health.

[47] Chamorro L.J., Alcaráz S., Sánchez-Oliva D., García-Calvo T., Torregrossa M. (2021). Fuelling the passion: Psychological needs and behavioural regulations as antecedents of passion for football. J. Sports Sci..

